# Dietary Selenium-Enriched Yeast Boosts Antioxidant Defense, Flesh Quality and Selenium Accumulation in Adult Common Carp (*Cyprinus carpio*)

**DOI:** 10.3390/antiox15091200

**Published:** 2026-09-19

**Authors:** Wanwen Chen, Scovia Kikoberwa, Jian Wu, Wu Jin, Xueyan Ma, Liufu Wang, Pao Xu, Hao Cheng, Haibo Wen

**Affiliations:** 1 Wuxi Fisheries College, Nanjing Agricultural University, Wuxi 214081, China; chenwanwen@ffrc.cn (W.C.);; 2 Key Laboratory of Integrated Rice-Fish Farming Ecology, Ministry of Agriculture and Rural Affairs, Freshwater Fisheries Research Center, Chinese Academy of Fishery Sciences, Wuxi 214081, China; 3 Sino-US Cooperative International Laboratory for Germplasm Conservation and Utilization of Freshwater Mollusks, Freshwater Fisheries Research Center, Chinese Academy of Fishery Sciences, Wuxi 214081, China; 4 State Key Laboratory of Food Science and Resources, School of Food Science and Technology, Jiangnan University, Wuxi 214122, China

**Keywords:** selenium-rich yeast, *Cyprinus carpio*, antioxidant defense, flesh quality, metabolic regulation

## Abstract

Selenium (Se) exerts essential biological functions via selenoprotein-mediated antioxidant defense and redox homeostasis. Selenium-enriched yeast (SEY) is a high-bioavailability organic Se source widely used in aquaculture. However, its effects on flesh quality in adult common carp (*Cyprinus carpio*) remain unclear. This study investigated the effects of graded dietary SEY on growth, muscle quality, Se deposition, antioxidant status, and associated metabolic responses in adult common carp. Results demonstrate that SEY supplementation (1.84 and 2.56 mg Se/kg) significantly improved growth performance without impairing physiological homeostasis. Mechanistically, SEY dose-dependently strengthened muscular antioxidant defense, with glutathione peroxidase activity increased by up to 7-fold, alongside elevated superoxide dismutase and catalase activities and reduced malondialdehyde content. This enhanced antioxidant capacity may maintain myofibrillar and membrane integrity, potentially contributing to the improvement of water-holding capacity and textural properties. SEY elevated umami amino acids and flavor nucleotides. Furthermore, in the 2.56 mg Se/kg supplemented group, SEY also increased muscle crude protein as well as organic Se deposition, with >99.99% of selenium present as selenocystine and selenomethionine. Untargeted metabolomics revealed that SEY reshaped the metabolome, enriching nucleotide metabolism and arginine biosynthesis pathways, which may partly account for the observed changes in umami-associated compounds. In conclusion, dietary SEY supplementation is an effective strategy for producing high-quality, Se-biofortified common carp.

## 1. Introduction

Aquatic products are recognized as a vital source of high-quality animal protein, polyunsaturated fatty acids, and essential micronutrients, playing an irreplaceable role in global food and nutrition security [1]. As one of the most extensively cultured freshwater species worldwide, common carp (*Cyprinus carpio* L.) contributes over 8% of China’s total freshwater output [2]. Despite its substantial economic value and favorable nutritional profile, intensively farmed common carp are chronically exposed to multiple stressors including high stocking density, deteriorated water quality, and pathogen challenge. These stressors trigger systemic oxidative stress, which disrupts myofibrillar protein structure and membrane integrity, ultimately leading to deteriorated flesh quality such as soft texture, impaired water-holding capacity, and reduced flavor and nutritional value [3]. Consequently, such quality deterioration has become a bottleneck limiting the sustainable development of the common carp industry. Therefore, developing green, safe, and effective nutritional strategies to enhance flesh quality is an urgent priority in food science and aquatic nutrition research.

Selenium (Se) is an essential trace element for both human and animal health, exerting its biological functions primarily through selenoproteins that regulate antioxidant defense, redox homeostasis, and metabolic health [4]. Selenium deficiency affects an estimated 15% of the global population. This public health issue is especially acute in China, where over 150 million people across 72% of the national territory are impacted [5]. Se biofortification of animal-derived food products has emerged as a safe and sustainable strategy to alleviate dietary Se deficiency [6]. Notably, fish act as efficient biological carriers for Se biofortification, accumulating highly bioavailable organic Se in edible muscle while simultaneously improving flesh quality via Se-mediated antioxidant regulation [7,8]. This dual benefit makes Se biofortification a promising approach to address both human Se malnutrition and aquaculture quality challenges.

Se sources utilized in aquaculture are primarily classified into inorganic forms (e.g., sodium selenite) and organic forms (e.g., selenium-enriched yeast, SEY). Although inorganic Se is cost-effective, its application is severely constrained by low bioavailability, a narrow safety margin, and potential toxicity at high doses. Conversely, SEY is a premium organic Se source widely adopted in the food and feed industries [9]. With selenomethionine as its primary functional component, SEY offers superior bioavailability, efficient tissue accumulation, and excellent biosafety, making it an ideal candidate for aquatic Se biofortification [10]. Previous studies have confirmed that SEY supplementation improves growth performance and antioxidant status in various juvenile fish species, including golden pompano (*Trachinotus ovatus*, 5.4–5.6 mg/kg Se requirement, initial body weight 8.9 ± 0.1 g) [11], largemouth bass (*Micropterus salmoides*, 1.5 mg/kg Se requirement, initial body weight 11.48 ± 0.43 g) [12], Meagre (*Argyrosomus regius*, above 3.0 mg/kg Se requirement, initial body weight 3.20 ± 0.17 g) [13], Triangular Bream (*Megalobrama terminalis*, 8.24 mg/kg Se requirement, initial body weight 4.26 ± 0.3 g) [14], zebrafish (*Danio rerio*) (3 mg/kg Se requirement, initial body weight 0.247 g ± 0.005) [15]. While the European Regulation (EC) No 1831/2003 stipulates a maximum supplemental limit of 0.5 mg Se/kg dry matter for animal feed, empirical evidence suggests that farmed fish often require higher inclusion levels to achieve optimal antioxidant capacity and health status, particularly when utilizing organic forms with higher digestibility and biological activity [16]. This discrepancy highlights the need for species-specific and developmental stage-specific evaluation of SEY supplementation in aquaculture.

However, current research predominantly focuses on the growth performance and physiological responses of juvenile fish. Systematic investigations into the regulatory effects of SEY on the edible quality attributes of market-sized adult common carp remain scarce. Flesh quality is a multifaceted trait encompassing physicochemical, nutritional, and sensory properties. Existing studies on Se-mediated quality regulation are still preliminary, often restricted to conventional texture parameters and proximate composition, while lacking comprehensive assessments of amino acid and fatty acid profiles, as well as flavor characteristics determined by free amino acids and nucleotides. Therefore, this study systematically investigated the effects of graded dietary SEY supplementation on growth performance, antioxidant capacity, Se deposition and speciation, and comprehensive flesh quality (including texture, WHC, nutritional composition, and flavor attributes) of adult common carp following a 56-day feeding trial. Furthermore, untargeted metabolomics was employed to elucidate the molecular regulatory mechanisms underlying SEY-mediated flesh quality improvement. The findings of this study will provide scientific data support for the application of SEY in the production of high-quality Se-enriched common carp, and lay a theoretical foundation for the development of functional aquatic products.

## 2. Materials and Methods

### 2.1. Ethics Statement

All animal experiments were conducted in accordance with the Guidelines for the Care and Use of Laboratory Animals and were approved by the Animal Care and Use Committee of the Freshwater Fisheries Research Center of the Chinese Academy of Fishery Sciences (LAECFFRC-2024-09-01).

### 2.2. Experimental Diets

The commercial basal diet was provided by Wuxi Tongwei Feedstuffs Co., Ltd. (Wuxi, China), and its ingredient composition is presented in Table S1. Selenium-enriched yeast (SEY; batch number: 20240113021) with a total selenium content of 2000 mg/kg based on the values provided by the manufacturer was purchased from Angel Yeast Co., Ltd. (Yichang, China). Four experimental diets were formulated by supplementing the basal diet with SEY at nominal selenium concentrations of 0, 0.5, 1.0, and 2.0 mg/kg, designated as the control, SEY-L, SEY-M, and SEY-H groups, respectively. Briefly, the basal diet was crushed, thoroughly mixed with SEY, extruded into pellets, air-dried at room temperature, and stored at −20 °C until use. The actual selenium concentrations in the finished diets were quantified via inductively coupled plasma mass spectrometry (ICP-MS). The detailed analytical procedures are provided in Section 2.9. The measured selenium levels of the four diets were 0.61 mg/kg (control), 1.15 mg/kg (SEY-L), 1.84 mg/kg (SEY-M), and 2.56 mg/kg (SEY-H).

### 2.3. Experimental Fish and Feeding Trial

Healthy common carp (*Cyprinus carpio*) were sourced from the Freshwater Fisheries Research Center, Chinese Academy of Fishery Sciences (Wuxi, China). Fish were acclimatized for 14 days prior to the feeding trial and fed a commercial basal diet without selenium-enriched yeast (SEY). Then, a total of 240 common carp were individually weighed and fish with an average body weight of 249.84 ± 1.27 g were randomly distributed into 12 net cages (2 × 2 × 1 m, L × W × H) at a stocking density of 20 fish per cage. Net cages were randomly assigned to four dietary treatments, with three replicate cages per treatment. During the formal feeding trial, fish were hand-fed to apparent satiation twice daily at 08:00 and 17:00. Uneaten feed residues from each cage were collected 30 min after every feeding event, then dried to constant weight for correction of actual feed consumption. Daily feed intake and fish mortality were recorded throughout the experimental period. Throughout the trial, water quality parameters were monitored twice weekly and maintained as follows: temperature 24.0 ± 2.0 °C, pH 7.0–8.0, dissolved oxygen > 7.5 mg/L, ammonia nitrogen < 0.5 mg/L, and nitrite < 0.05 mg/L.

### 2.4. Sample Collection

Following a 56-day feeding experiment, fish were fasted for 24 h. Thereafter, all surviving fish in each cage were counted and weighed to calculate survival rate (SR), weight gain rate (WGR), specific growth rate (SGR), and feed conversion ratio (FCR). Subsequently, twelve fish per dietary treatment (four fish randomly selected from each of the three replicate cages) were anesthetized with eugenol. Individual body weight, body length, liver weight, and visceral weight were recorded for each sampled fish to calculate the hepatosomatic index (HSI), viscerosomatic index (VSI), and condition factor (CF). Blood samples were collected from the caudal vein using heparinized syringes and centrifuged at 5000× *g* for 10 min at 4 °C to obtain plasma. For plasma biochemical parameters and antioxidant enzyme activities, individual samples from the 12 fish (4 fish per cage) were measured separately. The values from the four fish within each cage were then averaged to generate a cage-level mean (*n* = 3 cages per treatment) for statistical analysis (see Section 2.16). Dorsal muscle and liver tissues were rapidly dissected on ice. For biochemical and antioxidant analyses, tissues were snap-frozen in liquid nitrogen and stored at −80 °C. For proximate composition, amino acid, fatty acid, nucleotide compound, total selenium, and selenium speciation detection, muscle tissues from the four fish within each cage were fully homogenized and pooled into a single composite sample, yielding three pooled samples per treatment. Fresh dorsal muscle samples for texture profile analysis (TPA) and water-holding capacity (WHC) were obtained from six randomly selected fish per treatment. Individual measurements were averaged within each cage to obtain cage-level means, and statistical analysis was conducted based on three cage replicates (*n* = 3). Measurements were initiated immediately after sampling, and all analyses were completed within 24 h under refrigerated conditions (4 °C). For untargeted metabolomics analysis, dorsal muscle tissues from six randomly selected fish per treatment were collected and frozen at −80 °C.

### 2.5. Growth Performance and Morphometric Indices Calculations

At the end of the feeding trial, survival rate (SR), weight gain rate (WGR), specific growth rate (SGR), feed conversion ratio (FCR), hepatosomatic index (HSI), viscerosomatic index (VSI), and condition factor (CF) were calculated as follows [17].

Survival rate (SR, %) = numbers of final alive fish/numbers of initial fish × 100.

Weight gain (WG, %) = (final body weight − initial body weight)/initial body weight × 100.

Specific growth rate (SGR, %/day) = (ln final body weight − ln initial body weight) × 100/days.

Feed conversion ratio (FCR) = dry feed consumed/(final body weight − initial body weight).

Hepatosomatic index (HSI, %) = liver weight/final body weight × 100.

Viscerosomatic index (VSI, %) = viscera weight/final body weight × 100.

Condition factor (CF, %) = final body weight (g)/length (cm)^3^ × 100.

### 2.6. Muscle Proximate Composition Analysis

Muscle proximate composition was analyzed according to standard methods of the Association of Official Analytical Chemists [18]. Moisture content was measured via oven-drying to constant mass at 105 °C. Ash content was quantified after complete incineration in a muffle furnace at 550 °C for 12 h. Crude protein was detected using the Kjeldahl method with a nitrogen-to-protein conversion factor of 6.25. Crude lipid was extracted using a Soxhlet apparatus with petroleum ether.

### 2.7. Biochemical Assays

Triglycerides (TG), total cholesterol (TC), high-density lipoprotein cholesterol (HDL-C), and low-density lipoprotein cholesterol (LDL-C) were analyzed using a Roche Cobas 8000 c701 automatic biochemical analyzer (Roche, Mannheim, Germany).

### 2.8. Antioxidant Enzyme Activity Analysis

Frozen muscle and liver tissues were homogenized in pre-cooled saline (1:9, *w*/*v*), followed by centrifugation at 3000 rpm for 10 min at 4 °C to collect supernatants. The enzymatic activities of glutathione peroxidase (GSH-Px), catalase (CAT), and superoxide dismutase (SOD), as well as malondialdehyde (MDA) content, were determined using commercial assay kits (Nanjing Jiancheng Bioengineering Institute, Nanjing, China) according to the protocols. Protein concentrations were determined by the Bradford method to normalize enzyme activities.

### 2.9. Total Selenium Content and Selenium Speciation Analysis

For total Se quantification, approximately 0.2 g of muscle tissue was accurately weighed into a PTFE digestion vessel and digested with HNO_3_. A programmed heating procedure on a hot-plate digester was applied: 10 min from room temperature to 100 °C (hold 10 min); 10 min from 100 °C to 120 °C (hold 10 min); 10 min from 120 °C to 200 °C, followed by 60 min digestion at 200 °C. After digestion, solutions were concentrated, cooled, diluted with ultrapure water, and filtered through a 0.22 μm microporous membrane before ICP-MS (Thermo Fisher Scientific, Waltham, MA, USA) analysis. ICP-MS detection was performed in TQ-O_2_ mode with ^80^Se as the analyte isotope. ^159^Tb was used as the internal standard to correct for matrix effects. A series of standard solutions (1, 5, 10, 25, 50, and 100 μg Se/L) were prepared to establish the calibration curve. The linear regression equation was Y = 233.13X − 27.395, with a correlation coefficient (R^2^) of 0.9999. The limits of detection (LOD) and quantification (LOQ) were calculated based on signal-to-noise ratios (S/N) of 3 and 10, and were determined to be 0.03 and 0.10 μg/L, respectively. For accuracy and precision verification, a certified reference material (CRM, GBW10051, certified value: 1.54 ± 0.29 mg/kg) was analyzed. The measured value fell within the certified range, confirming the accuracy of the method. Precision was evaluated as the relative standard deviation (RSD, *n* = 3) of replicate analyses of the CRM, which was 1.94%.

For selenium speciation analysis, only muscle samples from the SEY-H group were detected. Selenium species were determined using enzymatic hydrolysis extraction coupled with HPLC-ICP-MS, following previously reported protocols [19,20]. Briefly, approximately 0.5 g of homogenized sample was accurately weighed and mixed with 15 mL Tris-HCl buffer (pH 8.5). Subsequently, 5% Alcalase, trypsin, and proteinase K were added to the mixture. The sample was incubated under magnetic stirring at 45 °C for 4 h, then centrifuged at 4000 rpm for 30 min. The resulting supernatant was filtered through a 0.22 μm filter membrane before HPLC-ICP-MS measurement. Mixed standard solutions containing selenocystine (SeCys_2_), selenomethionine (SeMet), methylselenocysteine (MeSeCys), selenite (Se^4+^), and selenate (Se^6+^) were prepared. Individual selenium species in sample extracts were identified by matching their retention times against those of corresponding reference standards. Selenium speciation data are now expressed as peak-area-normalized relative percentages. The relative proportion of each selenium species was calculated by dividing the chromatographic peak area of the individual target selenium species by the sum of peak areas of all five quantified selenium species, followed by multiplication by 100%.

### 2.10. Water-Holding Capacity Analysis

Water-holding capacity (WHC) was comprehensively evaluated by three indicators, including centrifugal loss, cooking loss, and thawing loss, with slight adjustments to previously published analytical procedures [21]. Specifically, centrifugal loss was defined as weight loss percentage after muscle centrifugation at 4000× *g* for 30 min at 4 °C. Cooking loss represented mass reduction after heating the muscle at 85 °C for 10 min. Thawing loss was determined by weighing sample weight change after −20 °C freezing for 24 h followed by complete thawing at room temperature. All loss values were expressed as weight percentage relative to initial fresh muscle weight.

### 2.11. Texture Profile Analysis and Shear Force Measurement

Texture profile analysis (TPA) was conducted on fresh dorsal muscle cubes (2 cm × 2 cm × 1 cm) using a TA-XT Plus texture analyzer (Stable Micro Systems, Ltd., Godalming, Surrey, UK) equipped with a P/5 cylindrical probe. The test was performed under the following conditions: pre-test speed of 2 mm/s, test speed of 1 mm/s, post-test speed of 2 mm/s, trigger force of 5 g, compression strain of 60%. Key textural indices including hardness, springiness, cohesiveness, gumminess, chewiness, and resilience were automatically obtained.

For shear force detection, muscle strips (2 cm × 3 cm × 1 cm) were measured on the identical texture analyzer equipped with an A/MORS shear blade. The test parameters were set as follows: a constant speed of 2 mm/s throughout pre-test, test, and post-test stages, a trigger force of 5 g, and a target compression deformation of 50%.

### 2.12. Free and Hydrolyzed Amino Acids Analysis

For free amino acids (FAAs) determination, muscle tissue was homogenized in 5% trichloroacetic acid solution, ultrasonically extracted, and centrifuged to collect supernatant. After filtration through a 0.22 μm organic membrane, the filtrate was analyzed on an S433D automatic amino acid analyzer (Sykam, Eresing, Germany). Quantified FAAs were further categorized into umami amino acids (UAAs), sweet amino acids (SAAs), and bitter amino acids (BAAs) to calculate respective cumulative contents alongside total free amino acids (TFAAs).

For hydrolyzed amino acids (HAAs) analysis, muscle samples were vacuum-sealed in glass ampules containing 6 mol/L HCl and hydrolyzed at 110 °C for 24 h. Post-hydrolysis neutralization and filtration were performed prior to instrumental analysis on the same amino acid analyzer. Total amino acids (TAAs), essential amino acids (EAAs), nonessential amino acids (NEAAs) and functional amino acids (FTAAs) were quantitatively determined.

### 2.13. Fatty Acid Composition Determination

Total lipids were extracted from muscle tissue using n-hexane/methanol mixed solvent (2:1, *v*/*v*). Transesterification was achieved via alkaline saponification with 0.5 mol/L methanolic NaOH followed by boron trifluoride-methanol-catalyzed fatty acid methylation. Derivatized fatty acid methyl esters were analyzed by a GC-2030AF gas chromatograph (Shimadzu, Kyoto, Japan). Individual fatty acid peaks were identified by matching retention times against certified mixed fatty acid standards, and results were expressed as relative percentage of total detected fatty acids. Atherogenic index (AI) and thrombogenic index (TI) were further computed using the empirical formulas described previously [22].

### 2.14. Muscle Nucleotide Quantification

Muscle tissues were homogenized in 10% (*w*/*v*) perchloric acid, centrifuged at 4000× *g* for 15 min at 4 °C, and the resulting supernatant was neutralized to pH 6.0–6.5 with 6 mol/L KOH. After centrifugation (10,000× *g*, 10 min, 4 °C) and 0.22 μm filtration, the final filtrate was subjected to high-performance liquid chromatography (HPLC; Waters e2695, Waters Co., Ltd., Milford, MA, USA) fitted with a C18 reversed-phase column to separate and quantify 5’-adenosine monophosphate (AMP), 5’-guanosine monophosphate (GMP) and 5’-inosine monophosphate (IMP).

### 2.15. Untargeted Metabolomics Analysis

Muscle metabolites were extracted from 100 mg of individual muscle tissue with methanol–water (4:1, *v*/*v*) supplemented with four internal standards (including 0.02 mg/mL L-2-chlorophenylalanine). Sample homogenization was performed via bead beating (Wonbio-96c, −10 °C, 50 Hz, 6 min), followed by low-temperature ultrasonication (5 °C, 40 kHz, 30 min). After incubation at −20 °C for 30 min, samples were centrifuged at 13,000× *g* for 15 min at 4 °C, and the supernatants were collected for UHPLC-MS/MS analysis.

Untargeted metabolomic profiling was conducted on a UHPLC-Q Exactive HF-X hybrid quadrupole-Orbitrap mass spectrometer (Thermo Fisher Scientific Inc., Waltham, MA, USA). Chromatographic separation was carried out on an ACQUITY HSS T3 column (100 mm × 2.1 mm i.d., 1.8 μm; Waters Corporation, Milford, MA, USA) at 40 °C. The mobile phases consisted of 0.1% formic acid aqueous solution (phase A) and 0.1% formic acid acetonitrile/isopropanol/water solution (phase B), with a constant flow rate of 0.40 mL/min. Mass spectra were acquired in both ESI^+^ and ESI^−^ modes with a scanning range of m/z 70–1050. The optimized MS parameters included ion spray voltages of 3500 V (ESI^+^) and −3000 V (ESI^−^), sheath gas of 50 arb, auxiliary gas of 13 arb, ion source temperature of 450 °C, and cyclic collision energies of 20–40–60 V for MS/MS fragmentation.

QC samples were prepared by equally mixing all sample extracts and inserted every 6 samples to monitor system stability. Raw data were preprocessed using Progenesis QI software (version 3.0, Waters Corporation, Milford, MA, USA), including peak alignment, retention time correction, and peak integration. Metabolite annotation was performed by matching MS and MS/MS spectral information against HMDB, METLIN, and MJDB databases. Data matrices were filtered using an 80% rule for missing values, normalized via total intensity normalization, and log10-transformed after removing features with RSD > 30% in QC samples. Multivariate statistical analyses, including orthogonal partial least-squares discriminant analysis (OPLS-DA), volcano plot visualization, and KEGG pathway enrichment, were implemented on the Majorbio Cloud Platform (https://cloud.majorbio.com). Differential metabolites were defined with thresholds of VIP > 1 and *p* < 0.05.

### 2.16. Statistical Analysis

Each net cage was defined as the biological experimental unit (*n* = 3 cages per treatment). Data represent the mean values of three replicate cages per group. Statistical analyses were conducted on these cage-level means using SPSS software (version 22.0; IBM Corp., New York, NY, USA). The Shapiro–Wilk test and Levene’s test were used to assess residual normality and homogeneity of variance, respectively. One-way analysis of variance (ANOVA) followed by Duncan’s post-hoc multiple-range test was used to detect inter-group significant differences. To statistically evaluate the linear and quadratic dose–response effects of dietary SEY, orthogonal polynomial contrasts were applied to the results from the Control, SEY-L, SEY-M, and SEY-H treatment. The Kruskal–Wallis H test, a non-parametric method, was used for metabolomic data to identify significantly altered metabolites. The significance threshold was set at *p* < 0.05 for all statistical comparisons.

## 3. Results and Discussion

### 3.1. Growth Performance

The growth performance of adult common carp fed diets supplemented with graded levels of selenium-enriched yeast (SEY) is summarized in Table S2. After the 56-day feeding trial, 100% survival was observed across all experimental groups. Medium (SEY-M, 1.84 mg Se/kg) and high (SEY-H, 2.56 mg Se/kg) SEY supplementation significantly improved weight gain rate (WGR) from 42.29 ± 2.27% (control) to 46.72 ± 1.21% and 50.18 ± 0.58%, respectively, and increased specific growth rate (SGR) from 0.58 ± 0.03%/day to 0.64 ± 0.01%/day and 0.68 ± 0.01%/day (*p* < 0.05). Additionally, feed conversion rate (FCR) for the SEY-M (1.89 ± 0.01) and SEY-H (1.79 ± 0.02) was significantly lower than for the control (1.95 ± 0.03) and SEY-L (1.98 ± 0.04) groups. Growth indicators kept rising across the gradient of measured total dietary selenium concentrations. Linear regression analyses further confirmed strong positive linear relationships between measured total dietary selenium concentration and both WGR (y = 38.871 + 4.289x, R^2^ = 0.834, *p* < 0.001) and SGR (y = 0.549 + 0.049x, R^2^ = 0.830, *p* < 0.001) (Figure S1). Growth performance continued to increase with increasing measured dietary selenium, and no saturation plateau was observed at the highest measured total Se concentration (2.56 mg Se/kg diet) in the present study. In contrast, hepatosomatic index (HSI), viscerosomatic index (VSI) and condition factor (CF) remained statistically unchanged among all treatments (*p* > 0.05).

The growth-promoting effects of dietary SEY observed in this study are consistent with previous reports in other aquatic species including juvenile meagre (*Argyrosomus regius*) and triangular bream (*Megalobrama terminalis*) [14,23]. As an essential micronutrient, selenium is a core component of selenoproteins that regulate antioxidant defense and cellular redox homeostasis. By alleviating endogenous oxidative stress, selenium may improve nutrient utilization efficiency and promote protein synthesis, which could partly contribute to accelerated growth performance [24]. The absence of significant changes in HSI, VSI, and CF aligns with observations in selenium-supplemented coho salmon (*Oncorhynchus kisutch*) [25]. The present results demonstrated that dietary SEY supplementation effectively enhanced growth performance of common carp, while exerting no significant effects on somatic indices (HSI, VSI, CF) under the conditions of this 56-day feeding trial.

### 3.2. Muscle Proximate Composition

**Figure 1:**
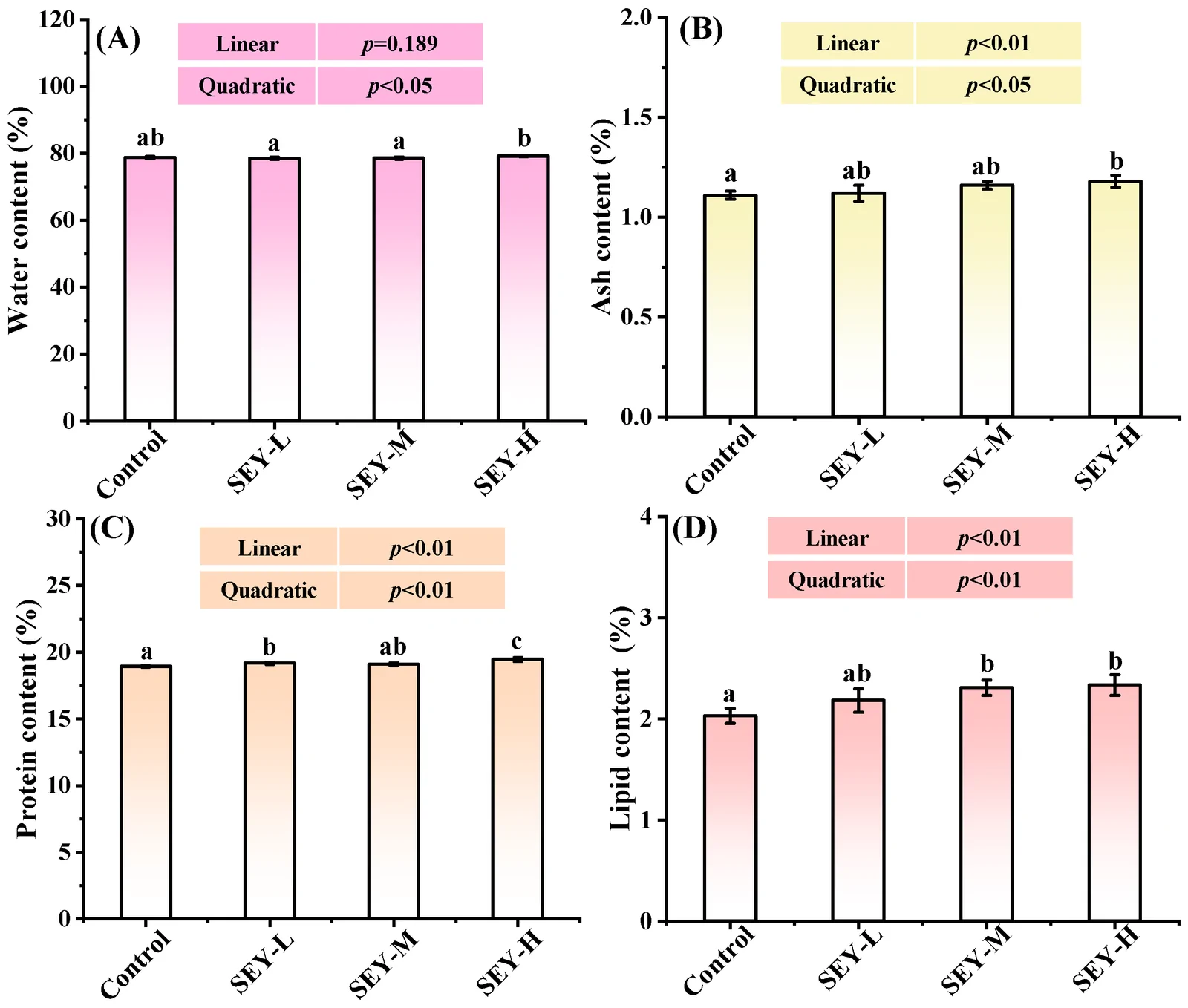
Proximate compositions of common carp (*Cyprinus carpio*) fed with different concentrations of selenium-enriched yeast for 56 days. (**A**) Water content, (**B**) Ash content, (**C**) Protein content, (**D**) Lipid content. Data are expressed as means ± SD (*n* = 3). Bars marked with different superscript letters are significantly different (*p* < 0.05). Linear (*p* < 0.05) or quadratic (*p* < 0.05) significance denoted a linear or quadratic influence of incremental dietary SEY levels on the index.

The proximate composition of common carp muscle is presented in Figure 1. Moisture content was relatively stable across groups, with a significant increase observed in the SEY-H group (79.18 ± 0.17%) compared with the control (78.77 ± 0.34%, *p* < 0.05). Ash content increased significantly in a dose-dependent manner with SEY supplementation, reaching the highest value in the SEY-H group (1.18 ± 0.03%, *p* < 0.05). Notably, dietary SEY also significantly elevated muscle crude protein content, which increased from 18.94 ± 0.06% (control) to 19.18 ± 0.09%, 19.10 ± 0.10% and 19.46 ± 0.15% in the SEY-L, SEY-M and SEY-H groups, respectively. Similarly, crude lipid content increased dose-dependently, with the SEY-H group showing a markedly higher value (2.33 ± 0.10%) than the control (2.03 ± 0.07%, *p* < 0.05).

The above changes align with the improved growth performance observed in Section 3.1, indicating that SEY supplementation may enhance nutrient assimilation and preferentially promotes deposition in skeletal muscle rather than visceral tissues. The elevated protein content is likely attributed to selenium-mediated upregulation of protein synthesis pathways and suppression of oxidative protein degradation, which supports previous findings in rainbow trout (*Oncorhynchus mykiss*) fed SEY-supplemented diets [26]. Similarly, the increase in muscle lipid is consistent with findings in triangular bream (*Megalobrama terminalis*), whereas protein content exhibited no significant difference [14]. The discrepancies may reflect species-specific differences in nutrient partitioning. The moderate elevation in muscle lipid content may contribute to improved flesh flavor and texture. The rise in ash content might reflect the enhanced mineral accumulation in muscle, specifically selenium (detailed in Section 3.5). Collectively, SEY supplementation may improve the nutritional profile of carp muscle by increasing protein, lipid, and mineral deposition.

### 3.3. Biochemical Indices

The effects of dietary SEY on lipid metabolism-related biochemical parameters are presented in Table S3. Compared with the control group, triglyceride (TG), total cholesterol (TC), and low-density lipoprotein cholesterol (LDL-C) levels exhibited non-significant downward trends, while high-density lipoprotein cholesterol (HDL-C) showed a significant increase in the SEY-H group (*p* < 0.05).

TG, TC, LDL, and HDL are well-established indicators of systemic lipid homeostasis in fish. The present findings are consistent with a previous study in common carp fed selenium-rich *Lactobacillus plantarum*, which also reported similar non-significant changes in these lipid parameters [27]. Notably, that study also demonstrated that selenium could alleviate high-salt-induced lipid metabolic disorders by reducing serum TG, TC, and LDL-C while increasing HDL-C levels. Our results indicated that dietary SEY supplementation at the tested dosages did not alter lipid-related plasma indices. However, the absence of significant changes in plasma lipid biomarkers, together with non-significant alterations in HSI, VSI, and CF values, cannot completely exclude potential adverse effects of high selenium intake, including possible long-term or subclinical toxic effects, owing to the limited 56-day feeding duration of this trial.

### 3.4. Antioxidant Capacity in Liver and Muscle

**Figure 2:**
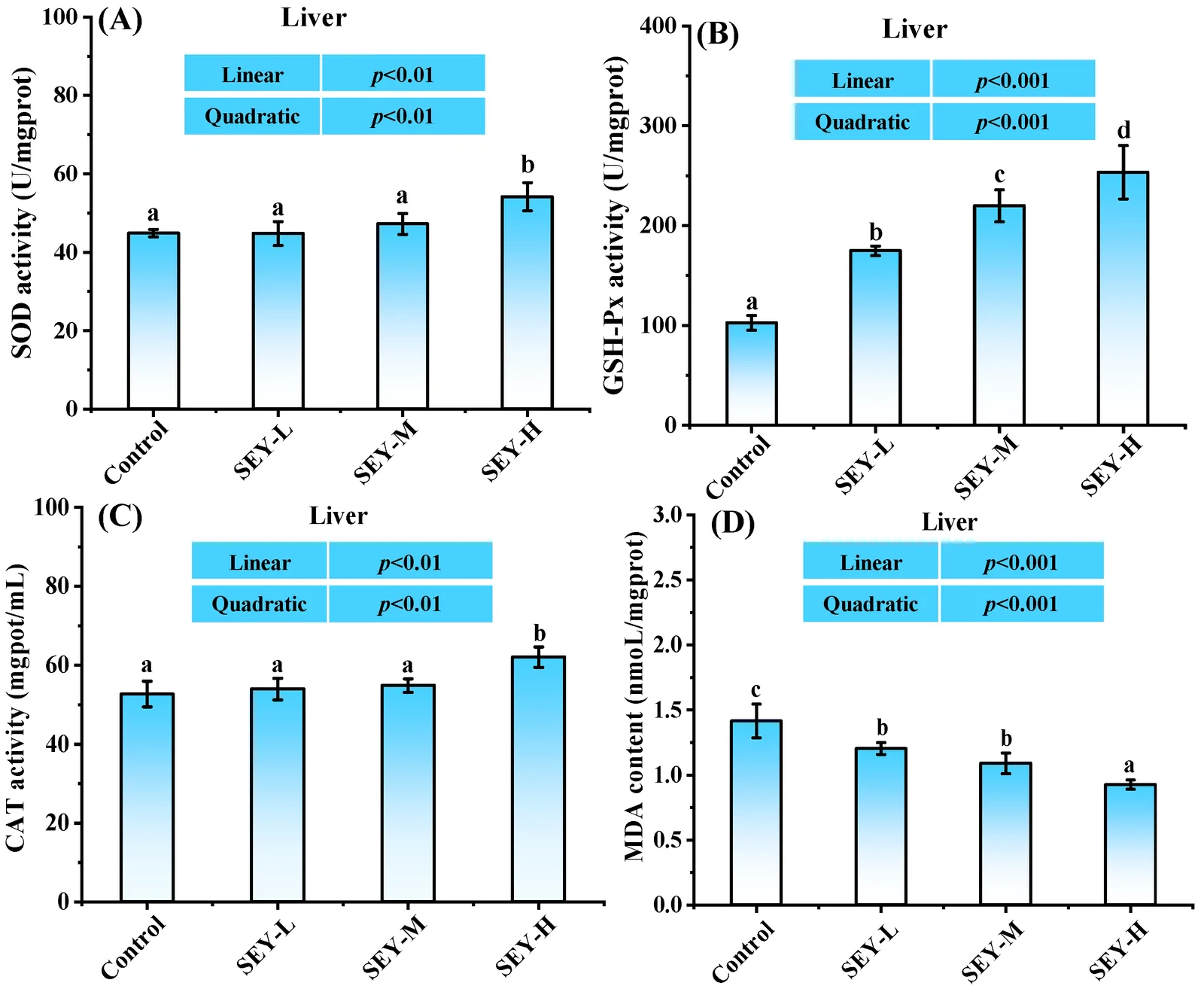

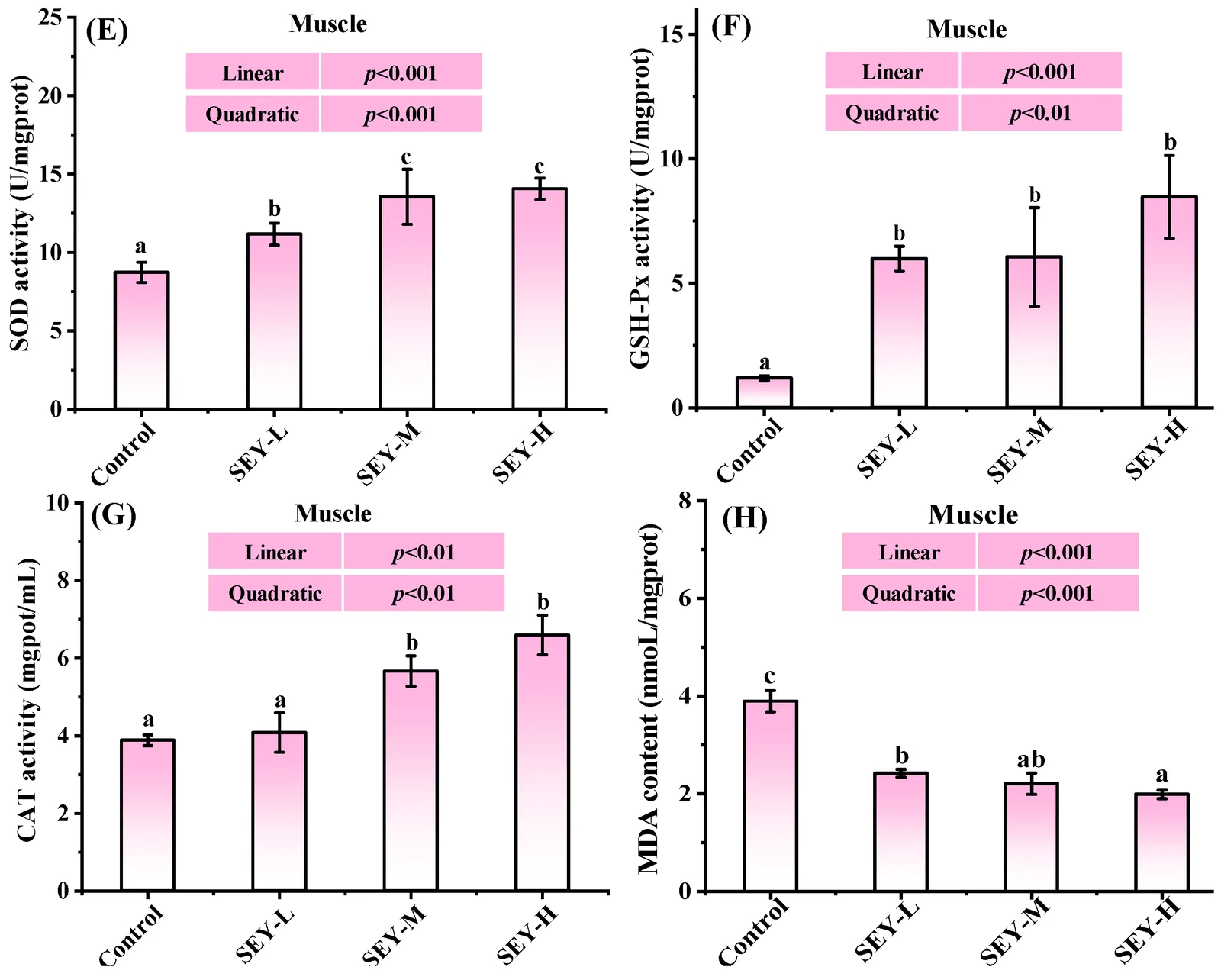
Effects of dietary selenium-enriched yeast (SEY) supplementation on antioxidant enzyme activities and malondialdehyde (MDA) content in liver and muscle tissues of adult common carp (*Cyprinus carpio*). (**A**) Superoxide dismutase (SOD) activity in liver, (**B**) Glutathione peroxidase (GSH-Px) activity in liver, (**C**) Catalase (CAT) activity in liver, (**D**) MDA content in liver, (**E**) SOD activity in muscle, (**F**) GSH-Px activity in muscle, (**G**) CAT activity in muscle, (**H**) MDA content in muscle. Data are expressed as means ± SD (*n* = 3). Bars marked with different superscript letters are significantly different (*p* < 0.05). Linear (*p* < 0.05) or quadratic (*p* < 0.05) significance denoted a linear or quadratic influence of incremental dietary SEY levels on the index.

The impact of dietary SEY supplementation on antioxidant enzyme activities and malondialdehyde (MDA) contents in liver and muscle tissues is presented in Figure 2. Hepatic glutathione peroxidase (GSH-Px) activity increased in a dose-dependent manner, with the SEY-H group reaching 2.47-fold that of the control. Both hepatic superoxide dismutase (SOD) and catalase (CAT) activities were significantly higher in the SEY-H group than in all other treatments (*p* < 0.05). Conversely, hepatic MDA content declined gradually with increasing dietary SEY levels, with the minimum value detected in the SEY-H group (*p* < 0.05).

In muscle tissue, GSH-Px activity was significantly higher in all SEY-supplemented groups than in the control, with the SEY-H group exhibiting a nearly 7-fold increase relative to the control (*p* < 0.05). Muscle SOD activity increased sequentially with elevated dietary SEY levels. Similarly, muscle CAT activity was significantly enhanced in the SEY-M and SEY-H groups compared with the control and SEY-L groups (*p* < 0.05). All SEY treatments significantly reduced muscle MDA content relative to the control, while no significant differences were observed among the SEY-supplemented groups.

It is well-known that GSH-Px, SOD and CAT act synergistically as the primary enzymatic defense system against reactive oxygen species [28]. In this study, dietary SEY supplementation remarkably upregulated the activities of these three antioxidant enzymes and reduced MDA accumulation in both liver and muscle tissues, consistent with previous findings in juvenile Catla catla [29], large yellow croaker (*Larimichthys crocea*) [30], and Nile tilapia (*Oreochromis niloticus*) [31]. The most pronounced elevation was observed in GSH-Px activity, which is directly explained by the fact that selenium is an indispensable constituent of the GSH-Px catalytic active center [32]. The coordinated increase in SOD and CAT activities further indicates that SEY may not only enhance selenoprotein-mediated antioxidant capacity but also activate non-selenium-dependent antioxidant pathways. This may help maintain cellular redox homeostasis and mitigate endogenous oxidative stress. The significant reduction in MDA content confirms that dietary SEY effectively suppresses lipid peroxidation in common carp tissues [33]. These robust antioxidant effects provide a potential mechanistic basis for the improved muscle quality observed in subsequent sections.

### 3.5. Muscle Selenium Accumulation and Speciation

**Figure 3:**
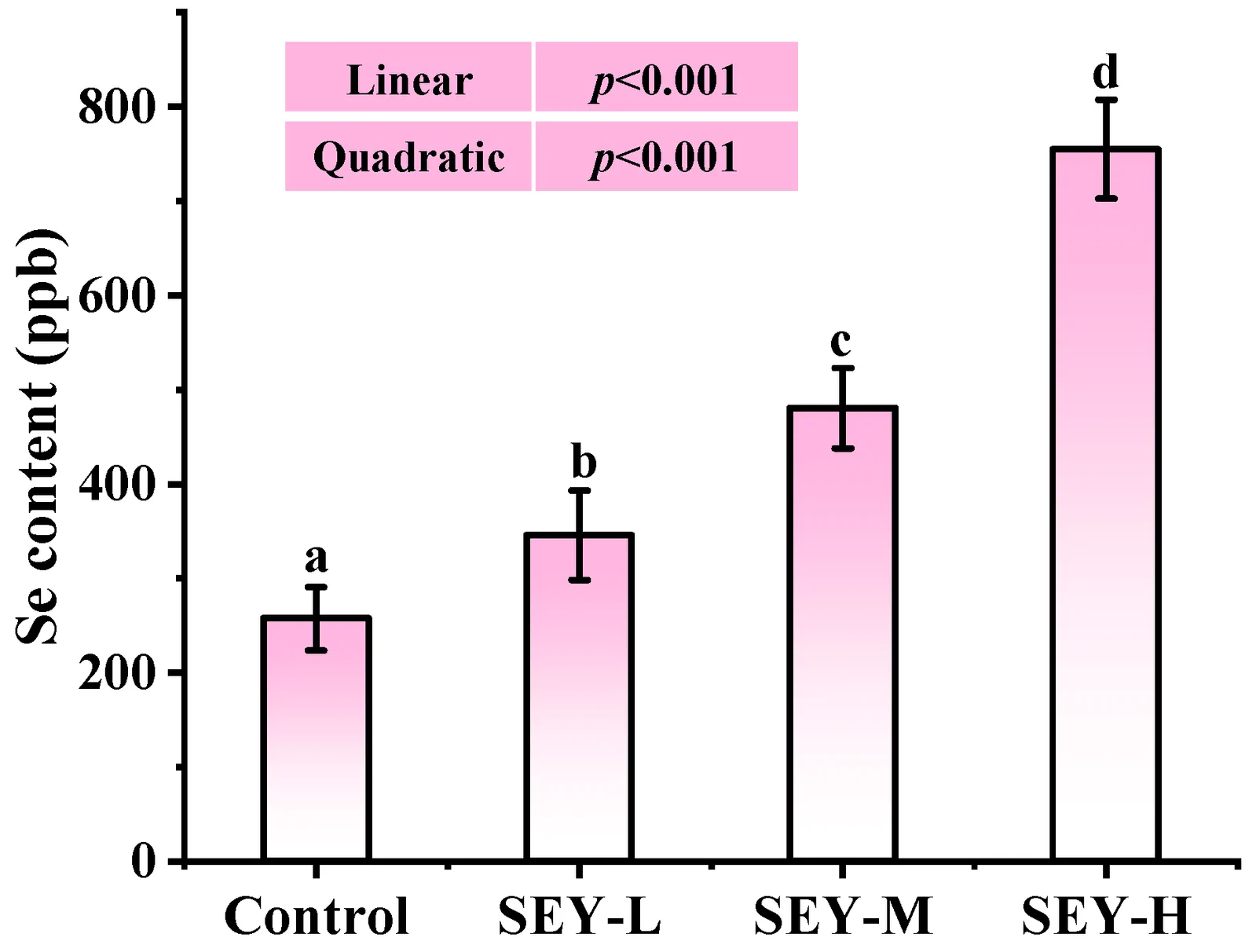
Selenium content in the muscle of common carp (*Cyprinus carpio*) fed with different concentrations of selenium-enriched yeast for 56 days. Data are expressed as means ± SD (*n* = 3). Bars marked with different superscript letters are significantly different (*p* < 0.05). Linear (*p* < 0.05) or quadratic (*p* < 0.05) significance denoted a linear or quadratic influence of incremental dietary SEY levels on the index.

Total selenium content in common carp muscle is presented in Figure 3. Dietary SEY supplementation significantly increased total muscle selenium concentration in a dose-dependent manner. The control group had the lowest selenium content (257.24 ± 33.66 μg/kg), which increased progressively to 345.67 ± 47.37 μg/kg (SEY-L), 480.52 ± 42.45 μg/kg (SEY-M) and reached a maximum of 754.96 ± 52.49 μg/kg (SEY-H), representing an approximately 3-fold increase in the SEY-H group relative to the control.

Selenium speciation analysis was performed on muscle samples from the SEY-H group. Five major selenium species were identified: selenocystine (SeCys_2_), selenomethionine (SeMet), methylselenocysteine (MeSeCys), selenite (Se^4+^) and selenate (Se^6+^). The good linear responses of the Se species are shown in Table S4. Based on peak-area normalization, organic selenium species accounted for more than 99.99% of total selenium-related chromatographic signal, with SeCys_2_ as the overwhelmingly predominant species (99.97 ± 0.01%), followed by SeMet (0.03 ± 0.01%). The remaining selenium-containing forms, including MeSeCys, Se^4+^, and Se^6+^, were detected at trace levels with relative proportions on the order of 10^−6^–10^−5^% and made negligible contributions to total selenium; therefore, these data are not presented herein. It must be emphasized that selenium speciation was only conducted in the SEY-H group. Thus, the observation of >99.99% organic selenium cannot be generalized to other dietary treatment groups. This trial primarily aimed to characterize the dominant selenium species accumulated in muscle under high-level SEY supplementation. Further multi-group speciation analysis is required to elucidate dose-dependent metabolic patterns of selenium in common carp muscle.

The dose-dependent increase in muscle selenium content indicates that organic selenium from yeast is efficiently absorbed and deposited in common carp muscle, consistent with previous reports in juvenile rainbow trout (*Oncorhynchus mykiss*) [34]. This finding also aligns with studies reported in *Onychostoma macrolepis*, demonstrating that organic selenium sources exhibit higher bioavailability than inorganic selenium, leading to greater selenium retention in fish tissues [35]. The nearly 3-fold higher selenium concentration in the SEY-H group suggests that dietary SEY supplementation represents an effective approach for producing selenium-enriched common carp.

Previous studies have reported varying selenium speciation profiles in fish muscle. For instance, in rainbow trout (*Oncorhynchus mykiss*) swim-up fry (initial average weight ~91 mg) fed SeMet-supplemented plant-based diets for 12 weeks, SeMet and SeCys together constituted approximately 76% of total selenium, with SeMet at ~50% and SeCys at ~26% [36]. This trend is largely consistent with our observation of SeCys_2_ and SeMet as the most abundant species in common carp muscle. However, in juvenile rainbow trout (200–300 g) treated with dietary SeMet for 2 weeks, the SeCys fraction was about 32–33% of total selenium in the muscles, while SeMet and SeCys were present in relatively greater amounts (≥30%) [37]. Such variations in selenium speciation profiles across studies can likely be attributed to biological factors such as fish species, developmental stage, dietary regimens, and feeding duration. In the present study, the high relative abundance of SeCys_2_ in the SEY-H sample can be attributed to the metabolic conversion of SeMet. As the major selenium species within SEY, SeMet can be incorporated into muscle proteins and further metabolized into SeCys_2_ [38]. The predominant deposition of organic selenium species (especially SeCys_2_) in SEY-H muscle may also be associated with the elevated GSH-Px activity in muscle tissue (Section 3.4), since seleno-amino acids serve as fundamental building blocks for functional selenoproteins [35,39,40]. Collectively, our results indicate that dietary SEY supplementation at 2.0 mg Se/kg promotes total selenium deposition and favors the accumulation of nutritionally relevant organic selenium forms in common carp muscle, which may improve the functional quality of carp muscle.

### 3.6. Texture Profile Analysis

The effects of dietary SEY supplementation on the textural properties of common carp muscle are presented in Table 1. Hardness values increased from 444.22 ± 17.15 g (control) to 532.71 ± 16.64 g, 545.19 ± 7.59 g, and 572.57 ± 36.67 g in the SEY-L, SEY-M, and SEY-H groups, respectively. Although a slight upward trend was observed with increasing SEY concentration, no significant differences were detected among the three SEY treatments (*p* > 0.05). Similarly, gumminess and chewiness were significantly elevated in all SEY groups compared with the control (*p* < 0.05). The SEY-L group exhibited the highest springiness value (0.88 ± 0.03), which was significantly higher than those of the SEY-M (0.82 ± 0.02), SEY-H (0.80 ± 0.02), and control (0.69 ± 0.02) groups (*p* < 0.05). Cohesiveness and resilience showed a similar dose-dependent decreasing trend within the SEY treatments. Additionally, shear force was significantly higher in the SEY-L group (4.32 ± 0.21 N) and SEY-M group (4.10 ± 0.32 N) than in the control group (3.48 ± 0.10 N) (*p* < 0.05). However, no significant differences were observed between the SEY-H groups and the control group (*p* > 0.05).

Enhanced texture parameters such as hardness and springiness are associated with firmer and more elastic flesh. Our findings further confirm that dietary SEY significantly improved the muscle textural quality of common carp, consistent with previous studies in grass carp (*Ctenopharyngodon idellus*) and largemouth bass (*Micropterus salmonides*) [41]. The improved textural properties can be primarily attributed to the enhanced antioxidant capacity induced by SEY supplementation (Section 3.4). Oxidative stress can trigger myofibrillar protein cross-linking and degradation, which may lead to softening and reduced elasticity of fish flesh. By scavenging ROS and suppressing lipid peroxidation, SEY might protect myofibrillar protein and cell-membrane status, which could partly explain the improved muscle texture observed in the present study [42]. Furthermore, selenium may be involved in the regulation of muscle protein synthesis and fiber development, possibly increasing myofibril density and contributing to higher shear-force values [43].

### 3.7. Water-Holding Capacity

**Figure 4:**
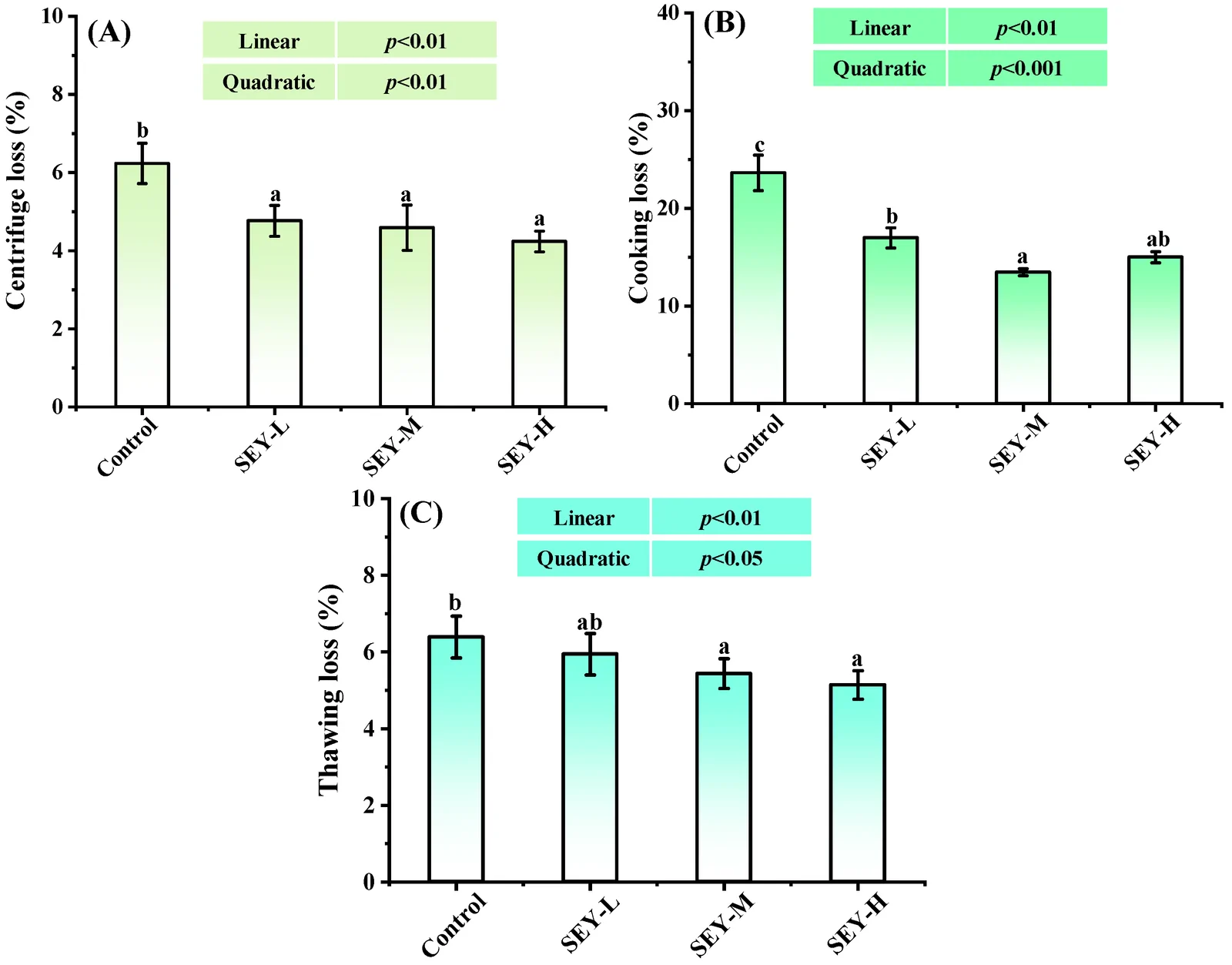
Water-holding capacity of common carp (*Cyprinus carpio*) fed diets supplemented with graded levels of selenium-enriched yeast for 56 days. (**A**) Centrifugal loss, (**B**) Cooking loss, (**C**) Thawing loss. Data are expressed as means ± SD (*n* = 3). Bars marked with different superscript letters are significantly different (*p* < 0.05). Linear (*p* < 0.05) or quadratic (*p* < 0.05) significance denoted a linear or quadratic influence of incremental dietary SEY levels on the index.

The effects of dietary SEY supplementation on muscle water-holding capacity (WHC) are presented in Figure 4. The control group exhibited the highest centrifugal loss (6.24 ± 0.52%), while SEY supplementation reduced centrifugal loss to 4.76 ± 0.39%, 4.59 ± 0.58% and 4.23 ± 0.27% in the SEY-L, SEY-M and SEY-H groups, respectively, with no statistically significant differences among the three SEY treatments (*p* > 0.05). Cooking loss also declined progressively with rising dietary SEY levels (Figure 4B), reaching 16.98 ± 1.04%, 13.46 ± 0.36% and 15.00 ± 0.56% in the SEY groups, all of which were significantly lower than the control group (23.63 ± 1.82%, *p* < 0.05). For thawing loss (Figure 4C), a numerical downward trend was observed from the control to the SEY-H group, although no significant intergroup differences were detected (*p* > 0.05). 

Lower water loss values indicate enhanced WHC, which corresponds to superior intracellular water retention and better meat quality. Our results demonstrate that dietary SEY has a pronounced positive effect on the WHC of common carp muscle, consistent with the improved textural properties described in Section 3.6. Similar beneficial effects of SEY on meat WHC have been reported in blunt snout bream (*Megalobrama amblycephala*) [44] and rainbow trout (*Oncorhynchus mykiss*) [26]. The improved WHC is also mechanistically linked to the enhanced antioxidant defense system (Section 3.4). ROS-induced oxidation of myofibrillar proteins may disrupt their three-dimensional hydrated network, potentially contributing to increased water loss. It is hypothesized that SEY supplementation could alleviate oxidative damage to proteins, which may help sustain the water-binding properties of muscle proteins and reduce centrifugal, cooking, and thawing losses. [45].

### 3.8. Amino Acid Profiles

The hydrolyzed amino acid composition of common carp muscle is presented in Table S5. A total of 17 amino acids were identified in all groups, with total amino acids (TAAs) showing a dose-dependent upward trend from 16.28 ± 0.57 g/100 g (control) to 17.96 ± 1.20 g/100 g (SEY-H), consistent with the increased crude protein content (Section 3.2). Due to the acid hydrolysis procedure triggering deamidation of asparagine (Asn) to aspartic acid (Asp) and glutamine (Gln) to glutamic acid (Glu), the combined pool of Asn + Asp is presented as Asx, and Gln + Glu is reported as Glx in Table S4. Glx, Asx, lysine (Lys) and leucine (Leu) were the predominant amino acids in carp muscle, consistent with previous reports in grass carp (*Ctenopharyngodon idellus*) [46], and all exhibited increasing trends with elevated SEY levels. Dietary SEY supplementation dose-dependently increased TAAs, essential amino acids (EAAs), non-essential amino acids (NEAAs), and functional amino acids (FTAAs), with the highest values observed in the SEY-H group. However, no statistically significant differences were detected in these cumulative amino acid indices among treatments (*p* > 0.05). Additionally, the EAA/NEAA ratio remained stable at 0.77–0.80 across all groups, indicating a well-balanced amino acid composition. These results demonstrate that SEY supplementation increases total muscle protein content while maintaining the proportional distribution of essential and non-essential amino acids, which is critical for the nutritional value of fish protein.

The free amino acid (FAA) composition of common carp muscle is shown in Table 2. Dietary SEY supplementation significantly modified the muscle FAA profile and enhanced accumulation of flavor-related amino acids. Total free amino acid (TFAA) content showed no significant differences among groups (*p* > 0.05), ranging from 0.5543 ± 0.0073 to 0.5800 ± 0.0155 g/100 g. However, total umami amino acids (Asp and Glu) increased significantly in a dose-dependent manner with SEY supplementation (*p* < 0.05), rising from 0.0153 ± 0.0016 g/100 g (control) to 0.0226 ± 0.0013, 0.0219 ± 0.0038 and 0.0233 ± 0.0036 g/100 g in the SEY-L, SEY-M and SEY-H groups, respectively. Glu, the primary contributor to umami taste, was elevated by 1.40-fold in the SEY-H group compared with the control, while Asp was increased by 1.97-fold. Although total sweet amino acids (SAAs) remained stable, glycine (Gly) content was significantly higher in the SEY-L and SEY-H groups relative to the control. In contrast, total bitter amino acids (BAAs) showed no significant differences among treatments (*p* > 0.05), while Lys content was significantly reduced in the SEY-H group (0.0176 ± 0.0062 g/100 g) compared with the control (0.0262 ± 0.0032 g/100 g, *p* < 0.05). The significant elevation in umami amino acids (Asp and Glu) across SEY treatments aligns with previous studies in *Paramisgurnus dabryanus* [47]. This improvement in the free amino acid profile is likely linked to selenium-mediated regulation of muscle protein turnover and redox balance. Importantly, the accumulation of potential flavor-related umami-relevant amino acids, together with elevated flavor nucleotides (Section 3.10), suggests possible synergistic flavor-modulating effects.

### 3.9. Fatty Acid Composition

The muscle fatty acid profiles of common carp fed graded levels of SEY are presented in Table S6. Across all treatment groups, unsaturated fatty acids (UFAs) constituted the dominant lipid fraction, accounting for approximately 79–80% of total fatty acids, while saturated fatty acids (SFAs) represented around 20%. No statistically significant differences were detected in total SFA and UFA contents between the control and SEY-supplemented groups (*p* > 0.05). However, total monounsaturated fatty acid (MUFA) content was significantly higher in the SEY-M group (31.18 ± 0.67%) compared with the control (29.07 ± 0.30%, *p* < 0.05), whereas total polyunsaturated fatty acid (PUFA) content was significantly reduced in this treatment group. The atherogenic index (AI) and thrombogenic index (TI), key indicators of the cardiovascular health potential of dietary lipids, exhibited downward trends with increasing dietary SEY inclusion, although these reductions did not reach statistical significance.

Despite the significant increase in total muscle lipid content (Section 3.2), the proportional distribution of SFAs and UFAs remained largely unchanged, suggesting that SEY supplementation promotes overall lipid deposition without altering the core fatty acid composition of carp muscle. Our findings are consistent with a previous report in red seabream larvae, where selenium-enriched rotifers did not alter whole-body fatty acid composition [48]. In contrast, organic selenium enrichment of Artemia markedly increased n-3 long-chain PUFAs, especially docosahexaenoic acid (DHA), in Asian sea bass larvae [49]. These divergent outcomes likely stem from multiple factors, including species-specific physiological responses to selenium, variations in selenium chemical form and dosage, and differences in experimental animal developmental stage and feeding regime [50]. The downward trend in AI and TI values further indicates that SEY supplementation may slightly improve the cardiovascular health benefits of carp muscle lipids.

### 3.10. Nucleotide Content Analysis

The effects of dietary SEY on muscle flavor-related nucleotides are summarized in Table 3. Dietary SEY supplementation significantly elevated the concentrations of adenosine-5’-monophosphate (AMP), guanosine-5’-monophosphate (GMP), and inosine-5’-monophosphate (IMP) in common carp muscle. AMP content increased from 7.02 ± 0.23 mg/100 g (control) to 7.57 ± 0.08 mg/100 g (SEY-H, *p* < 0.05). GMP content rose significantly from 77.57 ± 10.14 mg/100 g (control) to 98.70 ± 6.48 mg/100 g (SEY-M) and 94.22 ± 2.30 mg/100 g (SEY-H, *p* < 0.05). IMP content increased dose-dependently from 80.46 ± 14.13 mg/100 g (control) to 92.37 ± 7.52, 99.24 ± 8.38 and 111.25 ± 4.85 mg/100 g in the SEY-L, SEY-M and SEY-H groups, respectively, representing a 1.38-fold increase in the SEY-H group relative to the control (*p* < 0.05).

Nucleotides play a critical role in determining the taste and flavor of fish. IMP is the primary contributor to umami taste, while AMP acts as a flavor enhancer [45]. The concurrent increase in IMP and umami-relevant amino acids (Section 3.6) observed in the present study points to potential synergistic interactions among these potential flavor-related compounds in carp muscle of SEY-supplemented groups [21]. The increased nucleotide accumulation is further supported by the metabolomic analysis, which revealed significant enrichment of nucleotide metabolism pathways in the SEY-H group (Section 3.11).

### 3.11. Untargeted Metabolomic Analysis

**Figure 5:**
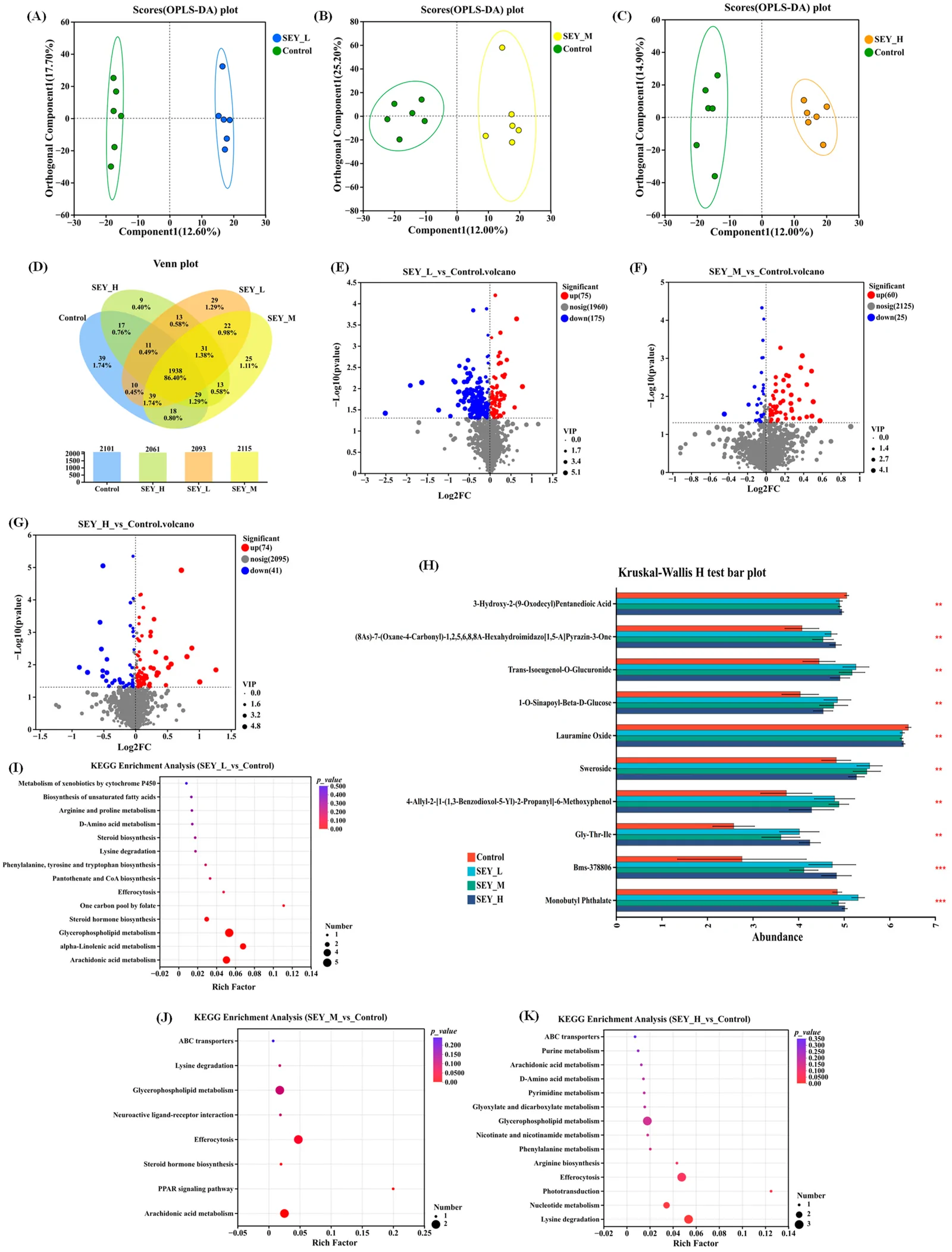
Untargeted metabolomic profiling of common carp muscle under dietary SEY supplementation. (**A**–**C**) OPLS-DA score plot. Ellipses indicate the 95% confidence interval of each group in the OPLS-DA score plots. (**D**) Venn diagram of shared and unique Differentially Accumulated Metabolites (DAMs) among the three treatment levels vs. Control. (**E**–**G**) Volcano plots of significantly regulated metabolites (SEY-L, SEY-M, and SEY-H vs. Control). Dashed lines represent the screening thresholds for significance. (**H**) Abundance profiles of core altered metabolites. Significance is denoted by asterisks: ** *p* < 0.01; *** *p* < 0.001. (**I**–**K**) KEGG functional enrichment of DAMs.

To elucidate the potential molecular mechanisms underlying SEY-mediated regulation of muscle quality and nutritional metabolism, untargeted metabolomic analysis was performed on common carp muscle samples. Orthogonal partial least-squares discriminant analysis (OPLS-DA) for pairwise comparisons between the control group and each SEY-supplemented group is shown in Figure 5A–C. Each pairwise OPLS-DA model was validated using seven-fold cross-validation and 200-iteration permutation testing. The models exhibited good explanatory power (R^2^Y = 0.981–0.995) with low Q^2^ intercepts (0.0016–0.167), indicating reliable model performance. Additionally, an unsupervised principal component analysis (PCA) including quality control (QC) samples was performed (Figure S2D), which showed tight clustering of QC samples and confirmed the stability of the metabolomics data. As shown in Figure 5D, a total of 1938 metabolites were commonly detected among the control and SEY-supplemented groups, accounting for 86.40% of the total identified metabolites, indicating that the majority of endogenous muscular metabolites maintained metabolic homeostasis upon SEY intervention. Pairwise differential screening identified distinct DAM profiles for each SEY dosage (Figure 5E–G). For SEY-L vs. Control, 250 DAMs were detected, including 75 upregulated and 175 downregulated metabolites. In SEY-M vs. Control, 60 upregulated and 25 downregulated metabolites were observed. As for SEY-H vs. Control, 74 metabolites were significantly upregulated and 41 downregulated.

The Kruskal–Wallis H test identified numerous significantly altered metabolites, among which the top ten signature metabolites are presented. Specifically, 4-Allyl-2-[1-(1,3-Benzodioxol-5-Yl)-2-Propanyl]-6-Methoxyphenol, trans-isoeugenol-O-glucuronide, sweroside, and Gly-Thr-Ile were significantly elevated, whereas 3-hydroxy-2-(9-oxodecyl) pentanedioic acid showed marked downregulation upon SEY supplementation. KEGG pathway enrichment analysis was performed to identify the key metabolic networks modulated by graded dietary SEY supplementation (Figure 5I-K). In the SEY-L vs. Control comparison, the most significantly enriched pathways were related to lipid and amino acid metabolism, including arachidonic acid metabolism, glycerophospholipid metabolism, alpha-linolenic acid metabolism, and steroid hormone biosynthesis. For the SEY-M group, arachidonic acid metabolism, PPAR signaling pathway, and steroid hormone biosynthesis were the top enriched pathways. The SEY-H group showed the most pronounced metabolic alterations, with nucleotide metabolism and lysine degradation as the core enriched pathways.

Notably, arachidonic acid metabolism and glycerophospholipid metabolism were ubiquitously enriched across all three SEY doses, representing the core conserved regulatory networks of organic selenium on fish muscle. Glycerophospholipids are the primary structural components of cell and myofibrillar membranes, while arachidonic acid is a key precursor of lipid peroxidation products [51]. The enrichment of these two pathways suggests that SEY-derived selenium may modulate antioxidant defense capacity. It has been reported that Se is an essential cofactor of glutathione peroxidase (GPx), which effectively scavenges reactive oxygen species (ROS) and inhibits phospholipid peroxidation, thereby stabilizing membrane ultrastructure [52]. This mechanistic finding provides molecular-level support for the improved antioxidant capacity, WHC, and textural properties observed in this study.

Distinct dose-specific metabolic responses were further observed. The PPAR signaling pathway was significantly enriched in the medium-dose SEY group, which may participate in the regulation of lipid homeostasis and antioxidant response [53]. Nucleotide metabolism exhibited prominent enrichment in the high-dose SEY group, which potentially explains the accumulation of IMP and GMP observed in the SEY-H group [54]. Collectively, the metabolomic results provide a comprehensive molecular framework explaining the multifaceted beneficial effects of SEY supplementation on common carp growth and flesh quality. However, the metabolomics findings should be considered exploratory and require independent validation through targeted assays. Further validation at the transcriptional or protein level is also required to confirm the inferred molecular regulatory pathways.

## 4. Conclusions

In conclusion, the present study systematically demonstrates that dietary supplementation with selenium-enriched yeast (SEY) effectively enhances growth performance and improves flesh quality in adult common carp (*Cyprinus carpio*). A dose-dependent increase in muscle crude-protein content and growth performance was observed among SEY-supplemented groups. Elevated activities of antioxidant enzymes, particularly glutathione peroxidase (GSH-Px), could be linked to the deposition of organic selenium species in muscle, which constitute key components of functional selenoproteins. A strengthened antioxidant defence system may suppress lipid peroxidation and myofibrillar protein oxidative injury, which might help sustain cell-membrane and myofibrillar-network status and further contribute to the observed changes in muscle textural properties and water-holding capacity (WHC). Untargeted metabolomics revealed that SEY regulates core metabolic pathways, including arachidonic acid and glycerophospholipid metabolism, as well as nucleotide metabolism and the PPAR signaling pathway. These pathways provide a molecular basis for the observed improvements in antioxidant status, nucleotide accumulation, and amino acid profiles.

Collectively, these findings suggest that SEY is a promising feed-additive candidate for producing high-quality, selenium-enriched common carp. Selenium-enriched carp may possess enhanced nutritional value and serve as a source of bioavailable selenium for human consumption, which could improve the nutritional and economic value of common-carp aquaculture. However, several constraints should be considered when interpreting our findings, including limited replication (*n* = 3 cages per treatment), the 56-day trial duration, absence of sensory evaluation, single-group selenium speciation analysis, and the correlative nature of metabolomics data lacking gene or protein validation. Future studies with expanded experimental designs and multi-level validation are required to verify these observations.

## Figures and Tables

**Table 1:** Texture profile analysis and shear force of common carp (*Cyprinus carpio*) fed diets supplemented with graded levels of selenium-enriched yeast for 56 days (mean ± SD, *n* = 3).

Parameters	Control	SEY-L	SEY-M	SEY-H	*p*-Value	
					Liner	Quadratic
Hardness	444.22 ± 17.15 ^a^	532.71 ± 16.64 ^b^	545.19 ± 7.59 ^b^	572.57 ± 36.67 ^b^	<0.001	<0.001
Springiness	0.69 ± 0.02 ^a^	0.88 ± 0.03 ^c^	0.82 ± 0.02 ^b^	0.80 ± 0.02 ^b^	0.129	<0.01
Cohesiveness	0.38 ± 0.01 ^a^	0.43 ± 0.01 ^b^	0.42 ± 0.01 ^b^	0.41 ± 0.01 ^b^	0.101	<0.01
Gumminess	168.14 ± 13.68 ^a^	224.35 ± 7.10 ^b^	207.84 ± 16.44 ^b^	211.36 ± 15.65 ^b^	0.075	<0.05
Chewiness	119.55 ± 8.48 ^a^	189.55 ± 7.77 ^b^	182.79 ± 7.42 ^b^	181.80 ± 6.30 ^b^	<0.05	<0.001
Resilience	0.22 ± 0.01 ^a^	0.28 ± 0.01 ^c^	0.26 ± 0.01 ^b^	0.25 ± 0.01 ^b^	0.327	<0.05
Shear force	3.48 ± 0.10 ^a^	4.32 ± 0.21 ^c^	4.10 ± 0.32 ^bc^	3.76 ± 0.30 ^ab^	0.571	<0.05

Note: Means in each row with different superscripts show significant differences (*p* < 0.05). Linear (*p* < 0.05) or quadratic (*p* < 0.05) significance denoted a linear or quadratic influence of incremental dietary SEY levels on the index.

**Table 2:** Free amino acid compositions of common carp (*Cyprinus carpio*) fed diets supplemented with graded levels of selenium-enriched yeast for 56 days (mean ± SD, *n* = 3).

Free Amino Acids (g/100 g)	Control	SEY-L	SEY-M	SEY-H	*p*-Value	
					Liner	Quadratic
Asp	0.0033 ± 0.0008 ^a^	0.0063 ± 0.0002 ^b^	0.0065 ± 0.0011 ^b^	0.0065 ± 0.0004 ^b^	<0.01	<0.01
Glu	0.0120 ± 0.0009 ^a^	0.0163 ± 0.0011 ^ab^	0.0154 ± 0.0030 ^ab^	0.0168 ± 0.0034 ^b^	0.059	0.112
Ser	0.0053 ± 0.0014	0.0044 ± 0.0040	0.0026 ± 0.0017	0.0044 ± 0.0028	0.507	0.555
His	0.2859 ± 0.0071	0.2917 ± 0.0190	0.3105 ± 0.0191	0.2933 ± 0.0074	0.331	0.287
Gly	0.1077 ± 0.0068 ^a^	0.1291 ± 0.0056 ^c^	0.1141 ± 0.0023 ^ab^	0.1189 ± 0.0026 ^b^	0.457	0.223
Thr	0.0138 ± 0.0001	0.0107 ± 0.0003	0.0116 ± 0.0025	0.0106 ± 0.0026	0.098	0.165
Arg	0.0104 ± 0.0040	0.0068 ± 0.0007	0.0085 ± 0.0004	0.0078 ± 0.0019	0.354	0.383
Ala	0.0212 ± 0.0002	0.0224 ± 0.0029	0.0208 ± 0.0014	0.0237 ± 0.0038	0.363	0.569
Tyr	0.0032 ± 0.0000	0.0033 ± 0.0005	0.0033 ± 0.0012	0.0020 ± 0.0014	0.167	0.170
Cys-s	0.0016 ± 0.0002	0.0025 ± 0.0014	0.0017 ± 0.0011	0.0017 ± 0.0008	0.888	0.751
Val	0.0037 ± 0.0000	0.0037 ± 0.0003	0.0037 ± 0.0003	0.0037 ± 0.0006	0.979	0.996
Met	0.0015 ± 0.0005	0.0015 ± 0.0000	0.0016 ± 0.0002	0.0016 ± 0.0003	0.722	0.937
Phe	0.0037 ± 0.0003	0.0033 ± 0.0007	0.0034 ± 0.0003	0.0033 ± 0.0005	0.407	0.617
Ile	0.0031 ± 0.0000	0.0032 ± 0.0002	0.0032 ± 0.0002	0.0029 ± 0.0007	0.503	0.622
Leu	0.0068 ± 0.0000	0.0070 ± 0.0000	0.0063 ± 0.0003	0.0062 ± 0.0001	0.111	0.290
Lys	0.0262 ± 0.0032 ^b^	0.0202 ± 0.0028 ^ab^	0.0235 ± 0.0030 ^ab^	0.0176 ± 0.0062 ^a^	0.067	0.205
Pro	0.0395 ± 0.0055	0.0355 ± 0.0069	0.0380 ± 0.0156	0.0277 ± 0.0045	0.178	0.356
Trp	0.0053 ± 0.0005	0.0056 ± 0.0004	0.0053 ± 0.0010	0.0056 ± 0.0014	0.783	0.965
UAAs	0.0153 ± 0.0016 ^a^	0.0226 ± 0.0013 ^b^	0.0219 ± 0.0038 ^b^	0.0233 ± 0.0036 ^b^	<0.05	<0.05
SAAs	0.1980 ± 0.0016	0.2089 ± 0.0145	0.1957 ± 0.0177	0.1932 ± 0.0095	0.418	0.491
BAAs	0.3410 ± 0.0039	0.3420 ± 0.0170	0.3624 ± 0.0227	0.3380 ± 0.0081	0.801	0.426
TFAAs	0.5543 ± 0.0073	0.5735 ± 0.0235	0.5800 ± 0.0155	0.5545 ± 0.0104	0.887	0.077

Note: FAAs, free amino acids; UAAs, umami amino acids, the total of Asp and Glu; SAAs, sweet amino acids, the total of Thr, Ser, Gly, Ala, Arg and Pro; BAAs, bitter amino acids, the total of Val, Met, Ile, Leu, Phe, Tyr, Lys, His, Trp, and Cys-s; TFAAs, total free amino acids. Data represent the mean values of three replicate cages per group. Means in each row with different superscripts show significant differences (*p* < 0.05). Linear (*p* < 0.05) or quadratic (*p* < 0.05) significance denoted a linear or quadratic influence of incremental dietary SEY levels on the index.

**Table 3:** Nucleotides of common carp (*Cyprinus carpio*) fed diets supplemented with graded levels of selenium-enriched yeast for 56 days (mean ± SD, *n* = 3).

Nucleotides (mg/100 g)	Control	SEY-L	SEY-M	SEY-H	*p*-Value	
					Liner	Quadratic
Adenosine-5’-monophosphate (AMP)	7.02 ± 0.23 ^a^	7.28 ± 0.28 ^ab^	7.38 ± 0.34 ^ab^	7.57 ± 0.08 ^b^	<0.05	0.059
Guanosine-5’-monophosphate (GMP)	77.57 ± 10.14 ^a^	86.42 ± 5.84 ^ab^	98.70 ± 6.48 ^b^	94.22 ± 2.30 ^b^	<0.01	<0.05
Inosine-5’-monophosphate (IMP)	80.46 ± 14.13 ^a^	91.95 ± 4.04 ^ab^	99.24 ± 8.38 ^bc^	111.25 ± 4.85 ^c^	<0.01	<0.01

Note: Data represent the mean values of three replicate cages per group. Means in each row with different superscripts show significant differences (*p* < 0.05). Linear (*p* < 0.05) or quadratic (*p* < 0.05) significance denoted a linear or quadratic influence of incremental dietary SEY levels on the index.

## Data Availability

The original contributions presented in this study are included in the article. Further inquiries can be directed to the corresponding authors.

## Supplementary Materials

The following supporting information can be downloaded at: https://www.mdpi.com/article/10.3390/antiox15091200/s1.
