# Supplementary material for: Dietary Selenium-Enriched Yeast Boosts Antioxidant Defense, Flesh Quality and Selenium Accumulation in Adult Common Carp (Cyprinus carpio)
Source: Antioxidants (Basel). 2026 Sep 19;15(9):1200. doi: 10.3390/antiox15091200 (PMC13603744; doi:10.3390/antiox15091200)
Supplement: Supplementary file 1 [file antioxidants-15-01200-s001.zip › antioxidants-4465529-supplementary.pdf]

Table S1 Composition of the commercial basal diet

Ingredients	Inclusion level (g)
Type 200 rapeseed meal	180
43% soybean meal	315
Peanut meal	45
Palm meal	90
Flour grade 1	60
Bran level 1	75
Spout corn husks	70
Domestic DDGS (1stgrade)	30
Rice bran	40
Soybean oil grade 3	5
Bentonite secondary	45
Calcium dihydrogen phosphate	18
Propionic acid type solid protection agent	0.3
Sodium chloride (96.5%)	2
L-ascorbate-2-phosphate	0.3
Polysaccharide complex premixed	1
Flavoring agent for aquatic products	1
L-catnitine hydrochloride (Liver cholesterin)	0.4
Vitamin premix feed	2
Microelement premix feed for omnivorous fish	20

Table S2 Growth performance of common carp (*Cyprinus carpio*) fed diets supplemented with graded levels of selenium-enriched yeast for 56 days (mean ± SD, $n = 3$).

Parameter	Control	SEY-L	SEY-M	SEY-H	*p*-value	
					Liner	Quadratic
IBW (g)	248.97±1.50	250.40±0.76	249.61±1.22	250.39±1.54	0.310	0.562
FBW (g)	354.24±4.69^{a}	357.37±3.45^{a}	366.22±2.58^{b}	376.04±3.69^{c}	<0.001	<0.001
WGR (%)	42.29±2.27^{a}	42.72±1.41^{a}	46.72±1.21^{b}	50.18±0.58^{c}	<0.001	<0.001
SGR (%/day)	0.58±0.03^{a}	0.59±0.02^{a}	0.64±0.01^{b}	0.68±0.01^{c}	<0.001	<0.001
FCR	1.95±0.03^{a}	1.98±0.04^{a}	1.89±0.01^{b}	1.79±0.02^{c}	<0.001	<0.001
HSI (%)	2.64±0.35	2.80±0.34	2.74±0.52	2.87±0.23	0.479	0.789
VSI (%)	20.01±3.88	21.47±4.36	23.76±4.14	25.05±2.84	0.079	0.233
CF (g/cm^{3})	1.49±0.11	1.52±0.12	1.54±0.03	1.55±0.05	0.337	0.637
Survival rate (%)	100	100	100	100	/	/

Note: IBW (g), initial body weight. FBW (g), final body weight. WG (%), weight gain rate. SGR (%/day), specific growth rate. FCR, feed conversion ratio. HSI (%), hepatosomatic index. VSI (%), viscerosomatic index. CF (%), condition factor. Means within the same row with different superscript letters are significantly different at $p < 0.05$. Linear ($p < 0.05$) or quadratic ($p < 0.05$) significance denoted a linear or quadratic influence of incremental dietary SEY levels on the index.

Table S3 Chemical index of common carp (*Cyprinus carpio*) fed with different concentrations of selenium-enriched yeast for 56 days (mean ± SD, *n* = 3).

Parameters (mmol/L)	Control	SEY-L	SEY-M	SEY-H	*p*-value	
					Liner	Quadratic
Triglycerides (TG)	2.53±0.23	2.38±0.11	2.35±0.05	2.45±0.36	0.638	0.536
Total cholesterol (TC)	3.00±0.07	2.92±0.07	2.90±0.20	2.98±0.21	0.794	0.617
High-density lipoprotein (HDL)	0.28±0.02[a]	0.31±0.01[b]	0.30±0.01[ab]	0.31±0.02[ab]	0.112	0.103
Low-density lipoprotein (LDL)	0.20±0.01	0.18±0.02	0.18±0.02	0.18±0.01	0.104	0.161

Note: Means in each row with different superscripts show significant differences ($p < 0.05$). Linear ($p < 0.05$) or quadratic ($p < 0.05$) significance denoted a linear or quadratic influence of incremental dietary SEY levels on the index.

Table S4 Linear regression equation and R^2, limit of detection (LOD), and limit of quantification (LOQ) of the method for detecting Se species.

Se species	Regression equation	Correlation coefficient	LOD (μg/L)	LOQ (μg/L)
$SeCys_2$	y=256.77x+53.22	0.9998	0.10	0.34
SeMet	y=306.63x+30.57	0.9996	0.15	0.51
MeSeCyS	y=217.15x-38.43	0.9999	0.12	0.41
Se (IV)	y=242.02x+36.07	0.9999	0.08	0.27
Se (VI)	y=281.00x-52.64	0.9997	0.09	0.30

Table S5 Hydrolyzed amino acid compositions of common carp (*Cyprinus carpio*) fed with different concentrations of selenium-enriched yeast for 56 days (mean ± SD, $n = 3$).

**Hydrolyzed amino acids (g/100 g)**	**Control**	**SEY-L**	**SEY-M**	**SEY-H**	***p*-value**	
					**Liner**	**Quadratic**
Asx	1.73±0.05	1.83±0.13	1.86±0.10	1.92±0.10	<0.05	0.110
Glx	2.73±0.08	2.85±0.29	2.92±0.18	3.08±0.25	<0.05	0.159
Ser	0.59±0.01	0.63±0.05	0.64±0.04	0.66±0.04	<0.05	0.140
His	$0.75±0.01^{a}$	$0.73±0.09^{a}$	$0.85±0.02^{b}$	$0.76±0.04^{a}$	0.415	0.481
Gly	0.92±0.01	0.91±0.05	0.89±0.03	0.92±0.07	0.964	0.787
Thr	0.69±0.02	0.74±0.06	0.76±0.05	0.77±0.04	<0.05	0.111
Arg	0.97±0.02	1.04±0.10	1.05±0.08	1.09±0.08	0.061	0.174
Ala	0.97±0.02	1.02±0.09	1.03±0.07	1.06±0.06	0.077	0.216
Tyr	0.46±0.00	0.50±0.06	0.51±0.05	0.52±0.04	0.092	0.206
Cys-s	0.06±0.00	0.07±0.01	0.07±0.01	0.07±0.01	0.119	0.134
Val	0.89±0.02	0.95±0.07	0.96±0.06	0.98±0.05	<0.05	0.110
Met	0.43±0.03	0.50±0.07	0.50±0.08	0.52±0.04	0.101	0.183
Phe	0.69±0.02	0.74±0.05	0.76±0.04	0.76±0.03	<0.05	0.082
Ile	0.81±0.02	0.87±0.07	0.88±0.06	0.90±0.05	<0.05	0.114
Leu	1.30±0.04	1.41±0.12	1.43±0.10	1.47±0.08	<0.05	0.098
Lys	$1.53±0.08^{b}$	$1.61±0.13^{ab}$	$1.70±0.08^{ab}$	$1.69±0.10^{a}$	<0.05	0.108
Pro	0.76±0.21	0.90±0.17	0.86±0.15	0.78±0.28	0.945	0.644
TAAs	16.28±0.57	17.32±1.39	17.69±1.05	17.96±1.20	0.063	0.160
EAAs	7.10±0.19	7.57±0.65	7.84±0.50	7.86±0.40	<0.05	0.102
NEAAs	9.18±0.39	9.75±0.76	9.84±0.56	10.10±0.82	0.095	0.243
FTAAs	1.09±0.04	1.16±0.09	1.18±0.07	1.20±0.08	0.071	0.177
EAA/NEAA	0.77±0.01	0.78±0.02	0.80±0.01	0.78±0.03	0.484	0.594

Note: TAAs, total amino acids. EAAs, essential amino acids. NEAAs, non-essential amino acids. FTAAs, functional amino acids. Means in each row with different superscripts show significant differences ($p < 0.05$). Linear ($p < 0.05$) or quadratic ($p < 0.05$) significance denoted a linear or quadratic influence of

incremental dietary SEY levels on the index.

Table S6 Fatty acids compositions of common carp (*Cyprinus carpio*) fed with different concentrations of selenium-enriched yeast for 56 days (mean ± SD, *n* = 3).

Fatty acids (%)	Control	SEY-L	SEY-M	SEY-H	*p*-value	
					Liner	Quadratic
C14:0	0.37±0.01[b]	0.33±0.02[a]	0.34±0.00[ab]	0.32±0.01[a]	<0.05	<0.05
C15:0	0.12±0.00	0.12±0.01	0.11±0.00	0.12±0.01	0.427	0.570
C16:0	14.76±0.01	15.38±0.41	14.77±0.19	14.80±0.49	0.654	0.422
C16:1	1.51±0.08[a]	1.85±0.05[bc]	2.01±0.08[c]	1.70±0.19[ab]	0.221	<0.01
C17:0	0.15±0.00	0.15±0.00	0.14±0.01	0.14±0.01	0.076	0.156
C17:1	0.11±0.00[a]	0.12±0.01[b]	0.12±0.00[b]	0.11±0.00[ab]	0.356	<0.05
C18:0	4.44±0.18	4.47±0.25	4.19±0.34	4.27±0.27	0.259	0.538
C18:1	25.67±0.15[a]	25.81±0.59[a]	27.73±0.90[b]	26.42±0.96[ab]	0.130	0.150
C18:2	34.97±0.17	33.83±1.09	34.43±1.04	34.55±0.98	0.787	0.488
C18:3n6	0.88±0.05	0.86±0.07	0.89±0.07	0.88±0.07	0.905	0.993
C18:3n3	2.22±0.04	2.13±0.16	2.31±0.15	2.23±0.14	0.589	0.868
C20:0	0.23±0.02	0.21±0.01	0.21±0.02	0.21±0.02	0.222	0.309
C20:1	1.69±0.08	1.43±0.13	1.42±0.14	1.45±0.22	0.097	0.076
C20:2	1.25±0.03[c]	1.11±0.05[b]	1.01±0.03[a]	1.12±0.05[b]	<0.05	<0.001
C20:3n6	2.74±0.06[b]	2.44±0.19[a]	2.25±0.22[a]	2.29±0.06[a]	<0.01	<0.01
C20:3n3	0.10±0.00[b]	0.09±0.00[a]	0.08±0.00[a]	0.08±0.01[a]	<0.05	<0.01
C20:4	5.07±0.38	5.69±0.35	4.70±0.29	5.59±1.11	0.769	0.915
C20:5	0.43±0.05	0.43±0.05	0.39±0.07	0.38±0.03	0.133	0.333
C22:0	0.09±0.02	0.07±0.00	0.07±0.00	0.08±0.01	0.518	0.302
C22:1	0.09±0.01	0.08±0.01	0.08±0.01	0.08±0.00	0.135	0.161
C22:6	3.11±0.11	3.42±0.45	2.75±0.47	3.20±0.48	0.740	0.919
∑SFA	20.16±0.21	20.74±0.66	20.04±0.22	19.80±0.67	0.234	0.216
∑UFA	79.84±0.21	79.26±0.66	79.96±0.22	80.20±0.68	0.234	0.218
∑MUFA	29.07±0.30[a]	29.18±0.36[a]	31.18±0.67[b]	29.71±1.36[ab]	0.182	0.187
∑PUFA	50.77±0.09[b]	50.08±0.32[b]	48.77±0.79[a]	50.49±0.71[b]	0.394	<0.05
UFA/SFA	3.96±0.05	3.83±0.15	3.99±0.06	4.05±0.17	0.221	0.208
AI	1.85±0.04	1.61±0.25	1.58±0.20	1.53±0.23	0.067	0.141
TI	0.78±0.02	0.81±0.04	0.75±0.01	0.76±0.05	0.277	0.508

Note: SFA, saturated fatty acid. UFA, unsaturated fatty acid. MUFA, monounsaturated fatty acids. PUFA, polyunsaturated fatty acids. AI, atherosclerosis index. TI, Thrombogenic index. Means in each row with different superscripts show significant differences ($p < 0.05$). Linear ($p < 0.05$) or quadratic ($p < 0.05$) significance denoted a linear or quadratic influence of incremental dietary SEY levels on the index.

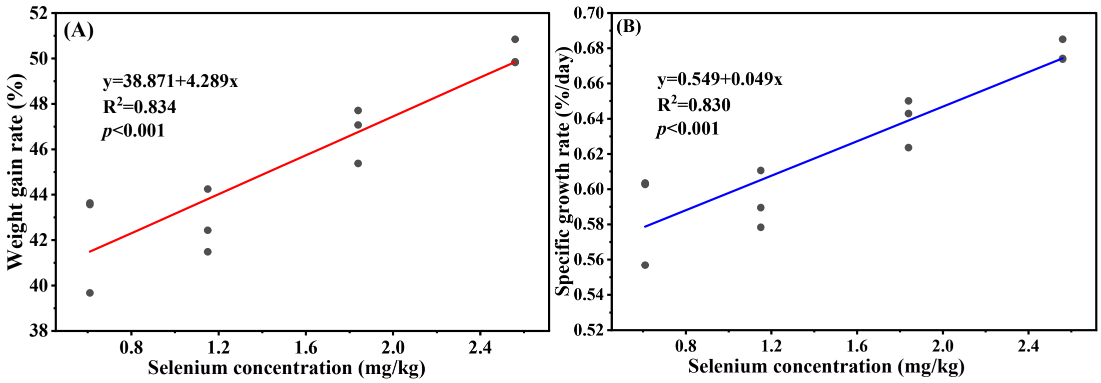

Figure S1. Linear-regression relationships between measured dietary selenium concentration and weight gain rate (WGR, A) and specific growth rate (SGR, B) of common carp.

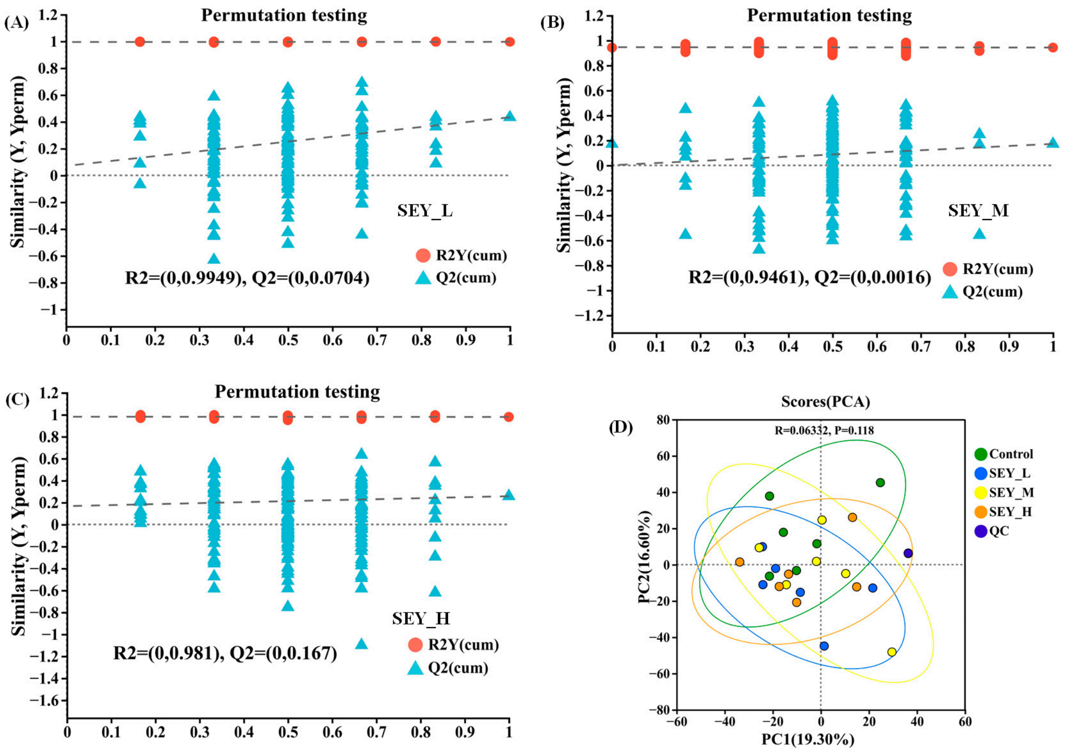

Figure S2. Permutation test results (n=200) for the OPLS-DA models comparing Control vs. SEY_L, SEY_M, and SEY_H groups (A–C) and unsupervised principal component analysis (PCA) for metabolomics data (D).
